# Retrospective Evaluation of Baseline Amino Acid PET for Identifying Future Regions of Tumor Recurrence in High-Grade Glioma Patients

**DOI:** 10.3390/cancers17121986

**Published:** 2025-06-14

**Authors:** Dylan Henssen, Michael Rullmann, Andreas Schildan, Stephan Striepe, Matti Schürer, Paola Feraco, Cordula Scherlach, Katja Jähne, Ruth Stassart, Osama Sabri, Clemens Seidel, Swen Hesse

**Affiliations:** 1 Department of Nuclear Medicine, University Hospital Leipzig, 04103 Leipzig, Germanyandreas.schildan@medizin.uni-leipzig.de (A.S.); matti.schuerer@googlemail.com (M.S.); osama.sabri@medizin.uni-leipzig.de (O.S.); swen.hesse@medizin.uni-leipzig.de (S.H.); 2 Department of Medical Imaging, Radboud University Medical Center, 6525GA Nijmegen, The Netherlands; 3 Institute for Neuroradiology, University Hospital Leipzig, 04103 Leipzig, Germany; stephan.striepe@uk-augsburg.de (S.S.); cordula.scherlach@medizin.uni-leipzig.de (C.S.); 4 Neuroradiology Unit, Santa Chiara Hospital, Azienda Provinciale per i Servizi Sanitari, 38100 Trento, Italy; paola.feraco@unitn.it; 5 Department of Neurosurgery, University Hospital Leipzig, 04103 Leipzig, Germany; katja.jaehne@medizin.uni-leipzig.de; 6 Institute of Neuropathology, University of Leipzig, 04103 Leipzig, Germany; ruth.stassart@medizin.uni-leipzig.de; 7 Department of Radiation Oncology, University Hospital Leipzig, 04103 Leipzig, Germany; clemens.seidel@medizin.uni-leipzig.de

**Keywords:** high grade glioma, invasion, tumor recurrence, amino acid PET

## Abstract

**Background/Objectives:** Positron emission tomography (PET) imaging with radiolabeled amino acids is increasingly used in glioma patients for biopsy planning, tumor delineation, prognostication, and therapy response assessment. This study investigated whether baseline amino acid PET imaging could identify regions at risk of future tumor recurrence. **Methods:** Retrospective case series of 14 patients with high-grade glioma. Contrast-enhanced magnetic resonance imaging (MRI) data of tumor recurrence and baseline imaging (PET-MRI) were co-registered. Volumes of interest (VOIs) of the high-grade glioma were derived from contrast-enhanced MRI at baseline and follow-up and from amino acid PET at baseline. The Dice similarity coefficient (DSC) was used to assess the overlap between VOIs. Furthermore, dynamic and static PET parameters were compared between the VOIs derived from contrast-enhanced MRI at follow-up and from the region of increased amino acid transport at baseline. **Results:** Regions of tumor recurrence in high-grade glioma patients overlap significantly more with baseline regions of increased amino acid transport on PET compared to regions of contrast enhancement on baseline MRI (*p* < 0.001). However, the static and dynamic PET statistics did not differentiate between regions that would later develop tumor recurrence and other areas of increased amino acid transport at baseline. **Conclusions:** These findings reaffirm the ability of amino acid PET to visualize the infiltrative components of gliomas not detected by contrast-enhanced MRI. Also, this study supports the role of amino acid PET in visualizing glioma infiltration beyond the MRI-visible tumor, but also indicates that accurately predicting the specific regions of recurrence based on baseline PET remains limited.

## Simple Summary

High-grade gliomas are aggressive brain tumors that often return after treatment due to their infiltrative growth. While MRI is commonly used to guide treatment, it fails to detect the full extent of tumor spread. This study investigated whether PET imaging with radiolabeled amino acids—performed before treatment—can predict where tumors will recur. In 14 patients, areas of future recurrence overlapped significantly with regions of increased amino acid uptake on baseline PET, more than with contrast-enhancing regions on MRI at baseline. However, specific PET parameters could not reliably distinguish which of these regions would later develop into recurrent tumors. These findings support the use of amino acid PET to visualize tumor infiltration beyond MRI-visible boundaries but highlight that more research is needed before it can predict recurrence with high precision.

## 1. Introduction

Adult-type diffuse glioma comprises a group of primary brain tumors that originate from glial cells (i.e., astrocytes or oligodendrocytes) that can be classified based on their molecular profile following recent recommendations by the World Health Organization (WHO) [1]. In contrast to other brain tumors, adult-type diffuse gliomas show an extensive, diffuse infiltration of tumor cells in the surrounding brain parenchyma over relatively long distances [2,3,4,5]. Due to this infiltrative growth, curative treatment is essentially impossible. Neuroimaging, especially magnetic resonance imaging (MRI), plays a pivotal role in glioma care. Different MRI techniques are used for the diagnosis and monitoring of tumor recurrence. Furthermore, MRI supports treatment decision making, guides the use of focused treatments, and determines the response to treatment [6,7]. However, conventional and advanced MRI techniques remain incapable of visualizing the infiltrative component of gliomas. In high-grade gliomas, blood–brain-barrier (BBB) deficiencies can be visualized by use of gadolinium-based contrast-agents. In general, these contrast-enhancing regions are known to contain the largest bulk of glioma cells. However, the invading glioma cells in the surrounding brain parenchyma cannot be observed on MRI [6,8,9]. The visualization of tumor spread is of crucial importance when treating patients suffering from glioma as patient survival depends strongly on the extent of tumor infiltration [10,11]. Despite surgical resection and an extended irradiated volume, a small portion of infiltrating glioma cells escape effective treatment and develop into tumor recurrence over time [12].

Next to MRI, the use of positron emission tomography (PET) imaging using radiolabeled amino acid tracers can play an important role in glioblastoma assessment [13,14]. Frequently, studies on the use of amino acid PET used either [^11^C]MET (L-(methyl-11C) methionine) or [^18^F]FET (18F-fluoro-ethyl-tyrosine), which enter the glioblastoma cell primarily through the lateral amino acid transporter (LAT) 1 and LAT2, respectively [15]. One of the advantages of amino acid PET imaging concerns the improved tumor delineation, as [^11^C]MET PET showed a significantly larger metabolically active tumor than the contrast enhancement region in glioma [16,17]. However, it remains unknown how this larger metabolically active tumor as visualized on PET imaging is related to the region of tumor recurrence in the post-treatment setting.

This study retrospectively investigated whether regions of tumor recurrence could be predicted using the pre-operatively obtained PET-MRI images using radiolabeled amino acid PET in high-grade glioma patients.

## 2. Materials and Methods

### 2.1. Ethical Approval

The local medical ethical committee approved the conduction of this study at our hospital (ethical review board assigned file number: 014/21-ek).

### 2.2. Patients

Patients older than 18 years, diagnosed with a high-grade adult-type diffuse glioma in the supratentorial brain who developed tumor progression, were eligible for inclusion. For inclusion, pre-treatment dynamic amino acid PET images (baseline) and pre-treatment contrast-enhanced, isotropic, T1-weighted MRI data (baseline) needed to be available. Furthermore, contrast-enhanced, isotropic, T1-weighted MRI data of the lesion reflecting tumor progression needed to be available. Furthermore, tumor recurrence was ensured through the histopathological assessment of tissue obtained through stereotactic biopsy. Patients with insufficient quality of MRI or PET data or the presence of extensive structural lesions other than the neuro-oncological disease (e.g., parenchymal loss due to (cortical) infarctions) were excluded. To confirm that contrast-enhancing lesions observed on follow-up MRI represented true tumor recurrence rather than progressive residual disease, immediate postoperative MRI scans (<72 h post-operation) were reviewed for all patients. Only new contrast-enhancing lesions not present on early postoperative imaging were considered indicative of recurrence.

The Picture Archiving and Communication System (PACS) of our university medical center was searched for patients who complied with the inclusion criteria between January 2013 and January 2023.

The demographic data of each included patient were extracted from the electronic patient file. These data consisted of the following: (1) age at diagnosis, (2) gender, (3) histopathological diagnosis at baseline, and (4) progression-free survival time.

### 2.3. PET-MRI Protocol

Contrast-enhanced, isotropic (1 mm) T1-weighted MRI data and dynamic radiolabeled amino acid PET-MRI data were acquired on a 3T PET-MRI system (*Siemens Biograph mMR, Siemens Healthineers, Erlangen, Germany*). The dynamic amino acid PET imaging protocol used either [^11^C]MET or [^18^F]FET. The radiosynthesis of the radiolabeled amino acids has been described in detail elsewhere [18]. After the injection of an intravenous bolus of the radiotracer, dynamic PET data were acquired in 3D list mode from 0 to 60 min. Emission recording reconstruction consisted of 38 time frames (time frames 1–12: 15 s each; time frame 13–19: 30 s each; time frame 20–24: 60 s each; time frame 25–29: 120 s each; time frame 30–34: 180 s each; time frame 35–36: 300 s each; time frame 37–38: 600 s each), covering the entire list-mode scan duration up to 60 min after injection. PET images were reconstructed using a 256 × 256 matrix, resulting in a voxel size 1.00 × 1.00 × 2.03 mm^3^ using a default subset expectation maximization algorithm with 8 iterations, 21 subsets, and a 3 mm Gaussian smoothing filter. For attenuation correction, the HiRES method was used as this method combines the individual Dixon attenuation correction approach with a bone attenuation template.

Post-treatment contrast-enhanced, isotropic (1 mm) T1-weighted MRI data, revealing the new contrast-enhancing lesion that was found to represent tumor progression, were recorded at a 3T MRI scanning system.

### 2.4. Co-Registration of Different Imaging Modalities at Different Time Points

Dynamic PET data were motion-corrected and co-registered with individual T1-weighted MRI images using PMOD (*PMOD Technologies LLC, Zürich, Switzerland*). Also, individual T1-weighted MRI images after treatment were co-registered to the pre-treatment individual T1-weighted MRI images using rigid image transformation and the image normalization tools available in PMOD (*PMOD Technologies LLC, Zürich, Switzerland)*. All registrations were controlled by one of the researchers (D.H., ten years of experience with (experimental) neuroimaging).

**Figure 1:**
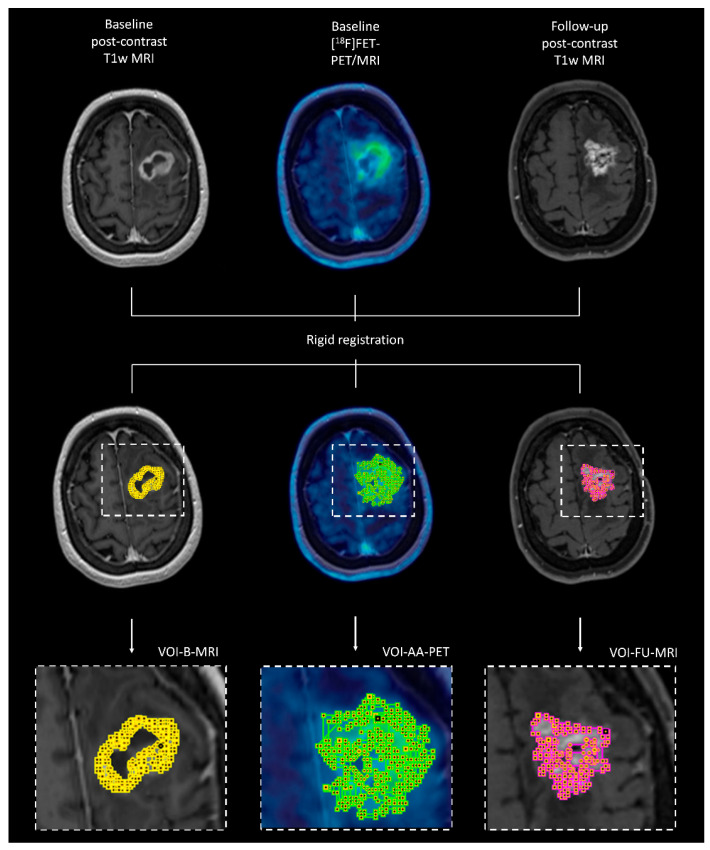
Schematic overview of the study methodology. Baseline contrast-enhanced T1-weighted MRI (left panel), baseline amino acid PET (in this example, [^1^⁸F]FET PET/MRI; middle panel), and follow-up contrast-enhanced T1-weighted MRI (in this example, a fat-suppressed T1-weighted sequence was used, right panel) were motion-corrected and co-registered using rigid image transformation. After alignment, three volumes of interest (VOIs) were segmented as follows: (1) the contrast-enhancing lesion on baseline MRI (VOI-B-MRI, yellow), (2) the region with increased amino acid uptake on baseline PET (VOI-AA-PET, green), and (3) the new contrast-enhancing lesion on follow-up MRI, representing histopathologically proven tumor recurrence (VOI-FU-MRI, pink). These VOIs were used to assess spatial overlap (Dice similarity coefficient) between imaging modalities and time points. Additionally, VOI-AA-PET and VOI-FU-MRI were used to create overlapping and non-overlapping subregions to investigate whether baseline static and dynamic PET parameters could predict future tumor recurrence.

The dynamic PET data of each patient were reconstructed as time-averaged images to create static PET reconstructions. Static images were reconstructed from the acquired images 20–40 min after injection of the radiotracer, following the current guidelines [14]. These static, time-averaged images were used for volume of interest (VOI) delineation using the “Hot 3D” semi-automatic segmentation method. This volume of interest covered the region of increased amino acid transport at baseline. The delineation of this region with increased amino acid transport at baseline was not influenced by the contrast enhancement, as observed on the T1-weighted MRI data. The same method of delineation was used to semi-automatically segment the contrast-enhancing lesions for each patient. The segmentation images were converted into binarized images that served as masks for further analyses. A schematic representation of the study methodology is provided in Figure 1.

### 2.5. Dice Similarity Coefficient

To predict whether regions of tumor recurrence overlapped with regions with altered amino acid transport, the co-registered masks of the region with increased amino acid transport were compared with the masks of the lesion reflecting tumor progression. Additionally, to predict whether regions of pathological contrast-enhancement at baseline overlapped with regions of future tumor progression, the co-registered masks of both MRI sessions were compared. The aforementioned comparisons of masks were carried out using the Dice similarity coefficient (DSC; see Equation (1)). The DSC was defined as a percentage (0–100%).

(1) $$DSC=\frac{2(X\cap Y)}{X\cup Y}$$

### 2.6. PET Statistics

The derived masks were also used for further dynamic PET analysis. Normalization of PET statistics was carried out by placing a 10 mm radius VOI in normal-appearing brain tissue containing both cortical gray matter and subcortical white matter [14]. Next to normalization (e.g., to calculate tumor-to-brain ratio; TBR), the VOI in the normal appearing brain tissue was used to ensure the time activity curves did not suffer from imaging artifacts. The different dynamic and static parameters derived from PET data were derived from PMOD. Time to peak (TTP in seconds) and area under the time activity curve (SUV*s) were the PET statistics derived from the dynamic PET imaging study. PET statistics derived from the static imaging study concerned the mean and maximum TBR.

### 2.7. Statistical Analysis

All statistical analyses were performed on IBM SPSS Statistics for Windows, version 28 (*IBM Corp., Armonk, NY, USA*). Patient characteristics were analyzed by applying descriptive statistics depending on the type and distribution of variables. A comparison of DSC values between the prediction of tumor recurrence was carried out using a paired Student’s T-test. Also, a paired Student’s T-test was used to compare the dynamic and static PET parameters of the VOIs of the contrast-enhancing region on follow-up MRI and the VOIs with increased amino acid transport on baseline PET. The level of significance for all hypothesis in this study was assumed at *p* < 0.05. Post hoc Bonferroni correction was applied when necessary.

## 3. Results

### 3.1. Patient Demographics

Fourteen patients (mean age 53.6 ± 4.1 years; 7 females) were included in this pilot study. Eleven radiolabeled amino acid investigations were carried out by use of [^11^C]MET with a mean administered dose of 711 ± 89 MBq. The remaining three studies were performed after a mean dose of 184 ± 25 MBq of [^18^F]FET. Histopathological diagnosis after biopsy or resection showed that three patients suffered from glioblastoma (*IDH*_wildtype_, all grade 4). Eight patients suffered from astrocytoma (*IDH*_mut_, 1p/19q intact, all grade 3) and three patients suffered from an oligodendroglioma (*IDH*_mut_, 1p/19q co-deleted, all grade 3). All patients underwent complete tumor resection. After surgery, a post-operative MRI was made within 72 h after surgery following international recommendations. Furthermore, all patients received adjuvant chemoradiotherapy. Patients suffering from astrocytoma or glioblastoma received adjuvant temozolomide. Oligodendroglioma patients received adjuvant PCV (procarbazine, lomustine and vinCRISTine). The median progression-free survival was found to be 12 months (ranging from 3 to 44 months). An overview of the patients included in this study is provided in Table 1.

### 3.2. Volumes of Interest on Baseline Amino Acid PET Yield Significantly Greater Overlap with Regions of Future Tumor Recurrence Compared to Baseline Contrast-Enhanced MRI

The median VOI size of the region with increased amino acid transport was significantly higher as compared to the median VOI size derived from baseline contrast-enhanced T1-weighted images (*Z =* −2.982; *p =* 0.003).

**Figure 2:**
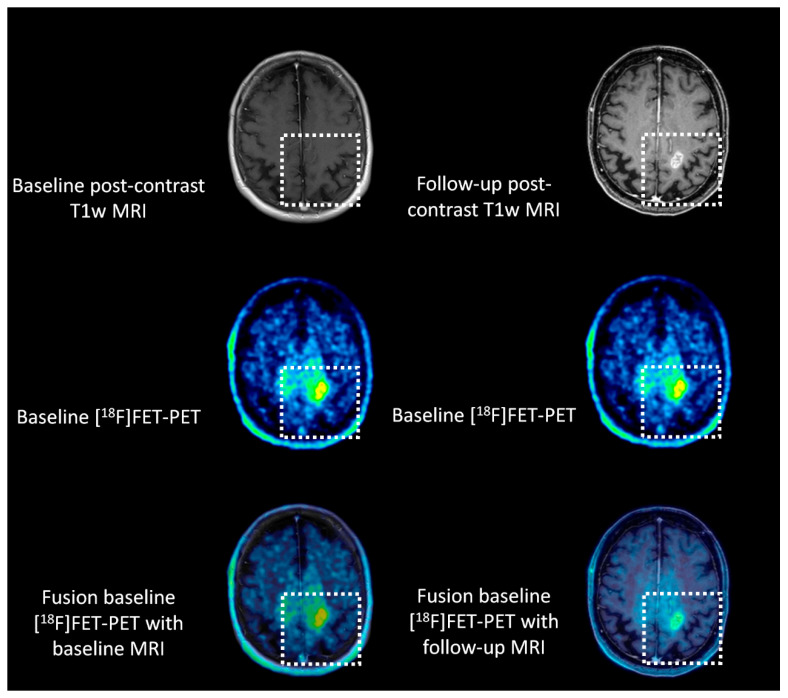
Exemplary glioblastoma case illustrating the predictive value of baseline [^18^F]FET-PET for future tumor recurrence. [^18^F]FET-PET/MRI at baseline can be observed in the first column. The second column shows a T1w MRI at follow-up (7 months after last radiation), the [^18^F]FET-PET at baseline, and the fused images. The first column shows a [^18^F]FET-PET/MRI section at the level of the centrum semiovale, with no abnormalities on T1w MRI, whereas the [^18^F]FET-PET image shows increased amino acid transport in the posteromedial part of the left frontal lobe. In the second column, post-contrast T1w MRI shows a new contrast-enhancing lesion (histopathologically proven tumor progression) in the posteromedial part of the left frontal lobe. The fusion with baseline [^18^F]FET-PET clearly indicates that the tumor recurrence at follow-up overlaps with the region of increased amino acid transport at baseline.

The mean overlap between the tumor recurrence masks and masks from regions with altered amino acid transport was shown to be 49.1 ± 8.6%. The mean overlap between tumor recurrence masks and baseline contrast-enhancement masks was significantly lower with 12.7 ± 5.7% (*t* = 4.291; *p* < 0.001). This suggests that, in general, amino acid PET at baseline covers regions of future tumor recurrence, whereas contrast-enhancement at baseline does not. An exemplary case is presented in Figure 2.

### 3.3. Baseline Static and Dynamic Amino Acid PET Statistics Cannot Be Used for Predicting the Region of Tumor Recurrence

When investigating if future regions of tumor recurrence could be recognized on baseline amino acid PET data (*n* = 11), no significant differences in dynamic PET statistics (i.e., time to peak and area under the time activity curve) were found between the VOIs containing the future regions of tumor recurrence on MRI and other regions with increased amino acid transport at baseline (*Z =* −0.421; *p =* 0.674 and *Z =* −1.690; *p =* 0.091, respectively). Furthermore, the TBRmax and TBRmean of the VOIs (n = 14) containing the future regions of tumor recurrence derived from the co-registered MRI data did not differ from the other regions with increased amino acid transport at baseline (*Z =* 1.125; *p =* 0.260 and *Z =* −0.889; *p =* 0.374, respectively). These results suggest that while baseline amino acid PET identifies a broader region of glioma infiltration—within which local tumor recurrence tends to occur—it does not allow for the accurate prediction of the specific subregions that will develop into tumor recurrence. This highlights the possible added value of amino acid PET for visualizing the at-risk area, albeit with limited specificity.

## 4. Discussion

This study shows that, after rigid image transformation and normalization, regions of tumor recurrence overlapped significantly more with baseline regions of increased amino acid transport on PET compared to regions of contrast enhancement on baseline MRI data. These findings suggest that the well-known invisible infiltrating tumor component can be visualized by use of PET imaging with radiolabeled amino acid tracers. Unfortunately, regions which develop into tumor recurrence are not different from other regions of increased amino acid transport when comparing dynamic or static PET parameters. Therefore, although regions of future tumor recurrence are found within the area with increased amino acid transport at baseline, the accurate prediction of which region will develop into a region of tumor recurrence remains elusive. Therefore, this study shows that, while baseline amino acid PET identifies a broader area containing future recurrence, it lacks the capacity to pinpoint which part of this area will progress.

During the last few years, the use of PET with radiolabeled amino acid tracers in glioma patients has been investigated extensively. In the pre-treatment setting, amino acid PET has been found to be promising for the non-invasive staging of WHO-grade gliomas. Characteristically, amino acid transport is higher in high-grade gliomas (WHO grade 3 and 4) as compared to low-grade gliomas (WHO grade 2), although uptake intensities vary and partially overlap [19,20,21,22]. The use of dynamic PET statistics has been found to be especially valuable with regard to non-invasive tumor grading [23,24]. Also, amino acid PET has been found useful for biopsy planning purposes [9]. More specifically, [^18^F]FET PET imaging was found to identify high-grade tumor regions within non-contrast-enhancing gliomas [13,25]. Next to the aforementioned, amino acid PET imaging has been found to be suitable for improving glioma geospatial distribution [13]. Various studies have validated the increased amino acid transport as observed on PET imaging with histopathological assessment of biopsied brain tissue [9,26,27]. Most recently, Song et al. showed that, in 31 glioma patients, [^18^F]FET-PET delineated significantly larger tumor volumes than contrast-enhanced MRI. Of the 21 biopsy samples obtained from regions with increased [^18^F]FET uptake, all were histopathologically confirmed as glioma tissue/infiltration [28]. As a result, the Response Assessment in Neuro-Oncology working group and the European Association for Neuro-Oncology recommendations advise to integrate amino acid PET in the work up to surgery in glioma patients. They state that the integration of amino acid PET into surgical planning allows the better delineation of the extent of resection beyond margins visible with contrast-enhanced MRI [13]. Taken together, the results of the current study fit the hypothesis that regions of future treatment recurrence are present at baseline. This has also been suggested by Arbizu et al., who found that amino acid PET imaging more accurately identified infiltrating regions of tumor extending beyond the MRI contrast-enhancing lesion. Therefore, amino acid PET helped in distinguishing regions with (infiltrating) tumor, non-tumoral edema, and normal brain tissue [16]. Indirect evidence supporting the hypothesis that regions of recurrence are present at baseline and can be identified by pre-treatment amino acid PET imaging was provided by Ort et al. [29]. They reported that, in a retrospective case series of thirty patients, overall survival was significantly longer (*p* = 0.006) when the gross total resection was based on amino acid PET images as compared to a gross total resection based on contrast-enhanced MRI [29].

To our knowledge, this study, for the first time, employs amino acid PET at baseline to investigate whether regions with increased amino acid transport will grow into regions of future recurrence in high-grade glioma patients. A particular strength of our approach concerns the use of rigid image transformation and normalization, ensuring robust data processing. Furthermore, the use of both dynamic and static PET statistics can be appreciated as a strength of the paper, although no significant differences were found between regions of future recurrence and other regions with increased amino acid transport. This study was limited by its retrospective, single-center design and small sample size. Future research should focus on the value of longitudinal amino acid PET data to investigate the spatiotemporal changes in residual regions with increased amino acid transport.

The current results illustrate a fundamental distinction as follows: while amino acid PET imaging detects a broader zone of glioma infiltration—within which recurrence tends to occur—it does not allow for pinpointing the exact subregion that will relapse. This means that amino acid PET is more sensitive than contrast-enhanced MRI for identifying at-risk tissue, but it lacks the specificity required for precise recurrence prediction. Clinicians should therefore view PET as a tool for improved treatment field delineation rather than as a predictor of recurrence at the voxel level.

## 5. Conclusions

This study demonstrates that regions of tumor recurrence in high-grade glioma patients overlap significantly more with baseline regions of increased amino acid uptake on PET than with contrast enhancement on baseline MRI. These findings confirm that amino acid PET imaging provides a more sensitive delineation of tumor infiltration. However, PET parameters—both static and dynamic—could not distinguish which subregions within the PET-positive area would eventually recur. Thus, amino acid PET should be considered a valuable tool for identifying a broader at-risk region that might benefit from intensified treatment, but not for the precise prediction of recurrence loci. Future research should aim to improve spatial prediction by combining PET with additional imaging modalities or molecular markers.

## 6. Key Points

Amino acid PET imaging reliably visualizes the infiltrative components of high-grade gliomas beyond what is seen on contrast-enhanced MRI.

Baseline amino acid PET indicates regions at risk of recurrence but cannot accurately predict which specific subregions will progress.

### 6.1. Pertinent Findings

This retrospective case series assessed whether baseline amino acid PET imaging can retrospectively identify regions that later develop into tumor recurrence in high-grade glioma patients. A significant spatial overlap was found between baseline areas of increased amino acid uptake and subsequent recurrence. These results underscore the utility of amino acid PET in delineating the full extent of glioma infiltration beyond MRI-visible margins, though its predictive specificity remains limited.

### 6.2. Implications for Patient Care

These findings may inform glioma treatment planning and underscore the need for further research to refine prediction models and improve outcomes in this aggressive disease.

## Figures and Tables

**Table 1:** Overview of the patients included in this study.

								Baseline Amino Acid PET				Lesion Derived from Follow-Up MRI			
*Nr*	*Age*	*Sex*	*Dose Tracer*	*Tumor Type*	*PFS* *(Months)*	*DSC Baseline Amino Acid PET* vs. *Follow-Up MRI*	*DSC Baseline MRI* vs. *Follow-Up MRI*	*TTP*	*Area Under the Time Activity Curve*	*TBRmax*	*TBRmean*	*TTP*	*Area Under the Time Activity Curve*	*TBRmax*	*TBRmean*
01	59	F	763 MBq [^11^C]MET	Astrocytoma (*IDH*_mut_, 1p/19q intact) grade 3	21	41.7%	8.6%	37.5	1980	2.7	3.8	52.5	2150	2.7	4.1
02	72	M	707 MBq [^11^C]MET	Astrocytoma (*IDH*_mut_, 1p/19q intact) grade 3	16	100.0%	0%	52.5	909	2.0	1.5	52.5	851	2.0	1.4
03	50	F	717 MBq [^11^C]MET	Astrocytoma (*IDH*_mut_, 1p/19q intact) grade 3	13	0%	0%	345	1120	1.7	1.5	157.5	1290	1.7	1.7
04	58	M	739 MBq [^11^C]MET	Oligodendroglioma (*IDH*_mut_, 1p/19q co-deleted) grade 3	11	33.3%	0%	90	2390	2.6	2.1	90	2920	2.5	2.6
05	46	M	448 MBq [^11^C]MET	Astrocytoma (*IDH*_mut_, 1p/19q intact) grade 3	8	27.5%	0%	157.5	1450	1.8	2.1	22.5	907	3.1	1.4
06	40	M	204 MBq [^18^F]FET	Astrocytoma (*IDH*_mut_, 1p/19q intact) grade 3	44	60.8%	0%	52.5	909	2.0	1.5	52.5	851	2.0	1.4
07	72	F	192 MBq [^18^F]FET	Glioblastoma (*IDH*_wildtype_) grade 4	12	85.6%	0%	37.5	1980	2.7	3.8	52.5	2150	2.7	4.1
08	56	F	749 MBq [^11^C]MET	Oligodendroglioma (*IDH*_mut_, 1p/19q co-deleted) grade 3	8	4.6%	0%	37.5	1920	3.0	1.5	127.5	1380	1.5	1.1
09	79	F	711 MBq [^11^C]MET	Oligodendroglioma (*IDH*_mut_, 1p/19q co-deleted) grade 3	12	38.1%	0%	52.5	1200	1.6	1.4	2250	822	1.4	1.0
10	36	M	750 MBq [^11^C]MET	Glioblastoma (*IDH*_wildtype_) grade 4	37	59.2%	37.8%	37.5	1180	4.9	1.6	37.5	978	2.9	1.3
11	37	F	738 MBq [^11^C]MET	Astrocytoma (*IDH*_mut_, 1p/19q intact) grade 3	12	78.5%	7.8%	157.5	1470	1.4	1.7	112.5	773	1.1	1.0
12	30	M	763 MBq [^11^C]MET	Astrocytoma (*IDH*_mut_, 1p/19q intact) grade 3	11	93.3%	68.5%	165	1970	1.1	2.0	60	1270	2.1	1.4
13	69	F	156 MBq [^18^F]FET	Glioblastoma (*IDH*_wildtype_) grade 4	10	14.0%	14.5%	82.5	1030	1.8	0.6	97.5	930	1.2	1.4
14	47	M	737 MBq [^11^C]MET	Astrocytoma (*IDH*_mut_, 1p/19q intact) grade 3	3	50.5%	40.3%	345	1120	1.7	1.5	157.5	1290	1.7	1.7

## Data Availability

The anonymized datasets generated during and/or analyzed during the current study are available upon reasonable request by contacting the corresponding author. The data are not publicly available due to their containing information that could compromise the privacy of the participants.

## Abbreviations

DSC	Dice similarity coefficient
MRI	Magnetic resonance imaging
PET	Positron-emission tomography
VOI	Volume of interest
[^11^C]MET	[S-methyl-^11^C]methionine
[^18^F]FET	O-(2-[^18^F]fluoroethyl)-L-tyrosine

## References

[1] Louis D.N., Perry A., Wesseling P., Brat D.J., Cree I.A., Figarella-Branger D., Hawkins C., Ng H.K., Pfister S.M., Reifenberger G. (2021). The 2021 WHO Classification of Tumors of the Central Nervous System: A summary. Neuro. Oncol..

[2] Scherer H.J. (1938). Structural Development in Gliomas. Am. J. Cancer.

[3] Giese A., Kluwe L., Laube B., Meissner H., Berens M.E., Westphal M. (1996). Migration of Human Glioma Cells on Myelin. Neurosurgery.

[4] Brat D.J., Castellano-Sanchez A.A., Hunter S.B., Pecot M., Cohen C., Hammond E.H., Devi S.N., Kaur B., Van Meir E.G. (2004). Pseudopalisades in glioblastoma are hypoxic, express extracellular matrix proteases, and are formed by an actively migrating cell population. Cancer Res..

[5] Brat D.J., Van Meir E.G. (2004). Vaso-occlusive and prothrombotic mechanisms associated with tumor hypoxia, necrosis, and accelerated growth in glioblastoma. Lab. Investig..

[6] Henssen D., Meijer F., Verburg F.A., Smits M. (2023). Challenges and opportunities for advanced neuroimaging of glioblastoma. Br. J. Radiol..

[7] Smits M. (2021). MRI biomarkers in neuro-oncology. Nat. Rev. Neurol..

[8] Lopez W.O., Cordeiro J.G., Albicker U., Doostkam S., Nikkhah G., Kirch R.D., Trippel M., Reithmeier T. (2015). Correlation of (18)F-fluoroethyl tyrosine positron-emission tomography uptake values and histomorphological findings by stereotactic serial biopsy in newly diagnosed brain tumors using a refined software tool. Onco Targets Ther..

[9] Pauleit D., Floeth F., Hamacher K., Riemenschneider M.J., Reifenberger G., Müller H.W., Zilles K., Coenen H.H., Langen K.J. (2005). O-(2-[18F]fluoroethyl)-L-tyrosine PET combined with MRI improves the diagnostic assessment of cerebral gliomas. Brain.

[10] Giese A., Bjerkvig R., Berens M.E., Westphal M. (2003). Cost of migration: Invasion of malignant gliomas and implications for treatment. J. Clin. Oncol..

[11] McGirt M.J., Attenello F., Gathinji M., Than K., Chaichana K.L., Datoo G., Olivi A., Weingart J.D., Brem H., Quinones-Hinojosa A. (2008). Extent of surgical resection is independently associated with survival in patients with malignant and low-grade brain astrocytoma. J. Neurosurg..

[12] Chiocca E.A., Silbergeld D.L., Piepmeier J.M., Laws E.R., Berger M.S. (2008). Extent of Surgical Resection Is Independently Associated with Survival in Patients with Hemispheric Infiltrating Low-Grade Gliomas Comments. Neurosurgery.

[13] Albert N.L., Weller M., Suchorska B., Galldiks N., Soffietti R., Kim M.M., la Fougere C., Pope W., Law I., Arbizu J. (2016). Response Assessment in Neuro-Oncology working group and European Association for Neuro-Oncology recommendations for the clinical use of PET imaging in gliomas. Neuro. Oncol..

[14] Law I., Albert N.L., Arbizu J., Boellaard R., Drzezga A., Galldiks N., la Fougère C., Langen K.J., Lopci E., Lowe V. (2019). Joint EANM/EANO/RANO practice guidelines/SNMMI procedure standards for imaging of gliomas using PET with radiolabelled amino acids and [(18)F]FDG: Version 1.0. Eur. J. Nucl. Med. Mol. Imaging.

[15] van der Kolk A.G., Henssen D., Schroeder H.W., Hall L.T. (2023). PET Agents for Primary Brain Tumor Imaging.

[16] Arbizu J., Tejada S., Marti-Climent J.M., Diez-Valle R., Prieto E., Quincoces G., Vigil C., Idoate M.A., Zubieta J.L., Penuelas I. (2012). Quantitative volumetric analysis of gliomas with sequential MRI and (1)(1)C-methionine PET assessment: Patterns of integration in therapy planning. Eur. J. Nucl. Med. Mol. Imaging.

[17] Suchorska B., Jansen N.L., Linn J., Kretzschmar H., Janssen H., Eigenbrod S., Simon M., Popperl G., Kreth F.W., la Fougere C. (2015). Biological tumor volume in 18FET-PET before radiochemotherapy correlates with survival in GBM. Neurology.

[18] Henssen D., Rullmann M., Schildan A., Striepe S., Schürer M., Scherlach C., Jähne K., Stassart R., Sabri O., Seidel C. (2024). The Diagnostic Accuracy of Dynamic Amino-Acid PET for the Differentiation Between Treatment Related Abnormalities and Tumor Progression in Post-Treatment Neuro-Oncology Patients.

[19] Rapp M., Heinzel A., Galldiks N., Stoffels G., Felsberg J., Ewelt C., Sabel M., Steiger H.J., Reifenberger G., Beez T. (2013). Diagnostic performance of 18F-FET PET in newly diagnosed cerebral lesions suggestive of glioma. J. Nucl. Med..

[20] Hutterer M., Nowosielski M., Putzer D., Jansen N.L., Seiz M., Schocke M., McCoy M., Göbel G., la Fougère C., Virgolini I.J. (2013). [18F]-fluoro-ethyl-L-tyrosine PET: A valuable diagnostic tool in neuro-oncology, but not all that glitters is glioma. Neuro. Oncol..

[21] Giammarile F., Cinotti L.E., Jouvet A., Ramackers J.M., Saint Pierre G., Thiesse P., Jouanneau E., Guyotat J., Pelissou-Guyotat I., Setiey A. (2004). High and low grade oligodendrogliomas (ODG): Correlation of amino-acid and glucose uptakes using PET and histological classifications. J. Neurooncol..

[22] Manabe O., Hattori N., Yamaguchi S., Hirata K., Kobayashi K., Terasaka S., Kobayashi H., Motegi H., Shiga T., Magota K. (2015). Oligodendroglial component complicates the prediction of tumour grading with metabolic imaging. Eur. J. Nucl. Med. Mol. Imaging.

[23] Pöpperl G., Kreth F.W., Mehrkens J.H., Herms J., Seelos K., Koch W., Gildehaus F.J., Kretzschmar H.A., Tonn J.C., Tatsch K. (2007). FET PET for the evaluation of untreated gliomas: Correlation of FET uptake and uptake kinetics with tumour grading. Eur. J. Nucl. Med. Mol. Imaging.

[24] Röhrich M., Huang K., Schrimpf D., Albert N.L., Hielscher T., von Deimling A., Schüller U., Dimitrakopoulou-Strauss A., Haberkorn U. (2018). Integrated analysis of dynamic FET PET/CT parameters, histology, and methylation profiling of 44 gliomas. Eur. J. Nucl. Med. Mol. Imaging.

[25] Kunz M., Thon N., Eigenbrod S., Hartmann C., Egensperger R., Herms J., Geisler J., la Fougere C., Lutz J., Linn J. (2011). Hot spots in dynamic (18)FET-PET delineate malignant tumor parts within suspected WHO grade II gliomas. Neuro. Oncol..

[26] Kracht L.W., Miletic H., Busch S., Jacobs A.H., Voges J., Hoevels M., Klein J.C., Herholz K., Heiss W.D. (2004). Delineation of brain tumor extent with [11C]L-methionine positron emission tomography: Local comparison with stereotactic histopathology. Clin. Cancer Res..

[27] Tripathi M., Sharma R., D’Souza M., Jaimini A., Panwar P., Varshney R., Datta A., Kumar N., Garg G., Singh D. (2009). Comparative evaluation of F-18 FDOPA, F-18 FDG, and F-18 FLT-PET/CT for metabolic imaging of low grade gliomas. Clin. Nucl. Med..

[28] Song S., Cheng Y., Ma J., Wang L., Dong C., Wei Y., Xu G., An Y., Qi Z., Lin Q. (2020). Simultaneous FET-PET and contrast-enhanced MRI based on hybrid PET/MR improves delineation of tumor spatial biodistribution in gliomas: A biopsy validation study. Eur. J. Nucl. Med. Mol. Imaging.

[29] Ort J., Hamou H.A., Kernbach J.M., Hakvoort K., Blume C., Lohmann P., Galldiks N., Heiland D.H., Mottaghy F.M., Clusmann H. (2021). (18)F-FET-PET-guided gross total resection improves overall survival in patients with WHO grade III/IV glioma: Moving towards a multimodal imaging-guided resection. J. Neurooncol..

